# Predictors of basic and instrumental activities of daily living among older adults with multiple chronic conditions

**DOI:** 10.1186/s12877-024-04947-w

**Published:** 2024-04-30

**Authors:** Azar Jafari-Koulaee, Eesa Mohammadi, Mary T. Fox, Aliakbar Rasekhi, Ozra Akha

**Affiliations:** 1 Department of Nursing, Faculty of Medical Sciences, Tarbiat Modares University, Tehran, Iran; 2 School of Nursing, Faculty of Health, York University Centre for Aging Research and Education, York University, Toronto, Ontario Canada; 3 Department of Biostatistics, Faculty of Medical Sciences, Tarbiat Modares University, Tehran, Iran; 4 Department of Endocrinology, Diabetes Research Center, Mazandaran University of Medical Sciences, Sari, Iran

**Keywords:** Multiple chronic conditions, BADL, IADL, Older adults

## Abstract

**Background:**

Understanding the predictors of functional status can be useful for improving modifiable predictors or identifying at-risk populations. Researchers have examined the predictors of functional status in older adults, but there has not been sufficient study in this field in older adults with multiple chronic conditions, especially in Iran. Consequently, the results of this body of research may not be generalizable to Iran. Therefore, this study was conducted to determine the predictors of functional status in Iranian older adults with multiple chronic conditions.

**Methods:**

In this cross-sectional study, 118 Iranian older adults with multiple chronic conditions were recruited from December 2022 to September 2023. They were invited to respond to questionnaires inquiring about their demographic and health information, basic activities of daily living (BADL) and instrumental activities of daily living (IADL), depression and cognitive status. The predictors included age, gender, marital status, education, number of chronic conditions, and depression. Descriptive and analytical statistical tests (univariate and multiple regression analysis) were used to analyze the data.

**Results:**

The majority of participants were married (63.9%) and women (59.3%). Based on the results of the multiple regression analysis, age (*B*=-0.04, *P* = 0.04), depression (*B*=-0.12, *P* = 0.04), and IADL (*B* = 0.46, *P* < 0.001) were significant predictors for functional status in terms of BADL. Also, marital status (*B*=-0.51, *P* = 0.05), numbers of chronic conditions (*B*=-0.61, *P* = 0.002), and BADL (*B* = 0.46, *P* < 0.001) were significant predictors for functional status in terms of IADL.

**Conclusion:**

The findings support the predictive ability of age, marital status, number of chronic diseases, and depression for the functional status. Older adults with multiple chronic conditions who are older, single, depressed and with more chronic conditions number are more likely to have limitations in functional status. Therefore, nurses and other health care providers can benefit from the results of this study and identify and pay more attention to the high risk older adult population.

## Introduction

Aging is a pervasive biological process that leads to a progressive and irreversible decline in physical function in all organ systems caused by the accumulation of damage in response to a variety of stressors [1]. Compared to other age groups, older adults are the most prone to have chronic diseases and disability [2]. More than 50% of the older adult population suffer from two or more chronic conditions or multiple chronic conditions [3, 4]. In Iran, the prevalence of multiple chronic conditions is increasing, and the burden caused by them in older adults is estimated at 90–92% [5]. It is estimated that between 2015 and 2035, the proportion of older adults with multiple chronic conditions will almost double (2015:9.8%; 2035:17.0%) [6]. Consequently, the growing older adult population with chronic conditions has become an important public health concern [7]. Multiple chronic conditions are associated with increased healthcare utilization [8], impaired mental health [9, 10], decreased quality of life [11] and increased functional limitations in performing basic activities of daily living (BADL) and instrumental activities of daily living (IADL) [12]. Studies conducted in different countries including China, Norway, Poland, and Spain have identified a significant relationship between multiple chronic conditions and functional status [13–16]. Functional status refers to a person’s capacity to perform basic and instrumental activities of daily living (BADL and IADL) [17]. The proportion of older adults experiencing limitations in their capacity to perform BADL and IADL differs significantly in different parts of the world. For example, in one study in Turkey, it was reported that 11.2% of older adults were limited in BADL and 45.8% were limited in IADL [18] whereas in Saudi Arabia, the prevalence of ADL and IADL disability in older adults has been estimated at 24.6% and 58.5%, respectively [19]. In Iran, the prevalence of BADL and IADL disability in older adults has been estimated at 2.5% and 48.2%, respectively [20]. Despite the existence of studies on functional status especially in developed countries, no studies have examined the predictors of functional status in older adults with multiple chronic conditions in Iran. Compared to developed countries, Iran has different health and social support systems policies especially for older adults, in which a number of them were not successful [21]. Social support is recognized as an important social determinant of health, as it helps people meet their physical and emotional needs and reduces the effects of stressful events on their lives [22], therefore, the lack of suitable support systems for older adults can affect the level of their functional status. In addition, in most developed countries, policies are aimed at providing geriatric services to older adults at home and community-based services to cover a wider range of services and also to empower older adults to live independently in society [23]. Meanwhile, in Iran, community-based geriatric services, as well as the policy of providing integrated services, have quantitative and qualitative defects especially in implementation process [24]. The most important obstacles to the implementation of general health policies in Iran were the lack of formulated strategies, poor management, lack of a comprehensive national action plan, minimal information infrastructure, and insufficient funding [25]. Consequently, due to the difference in health and support system policies especially for older adults in Iran compared to other countries, the levels of functional status or ability to perform basic and instrumental activities of daily living can be different in older adults, therefore, the results in other countries may not be generalizable to Iran. In addition, one of the important goals in the health care of patients with multiple chronic conditions is to improve their functional status, and to achieve this goal, it is necessary to understand the predictors of functional status because some predictors may be modifiable and thus may be treatable. Also, there may be some unmodifiable predictors that can be used to identify at-risk populations. In addition, understanding the predictors of functional status can guide the development of healthcare programs targeting older adults with multiple chronic conditions [26]. Therefore, the present study was conducted to determine the predictors of functional status in Iranian older adults with multiple chronic conditions.

## Conceptual framework

**Fig. 1:**
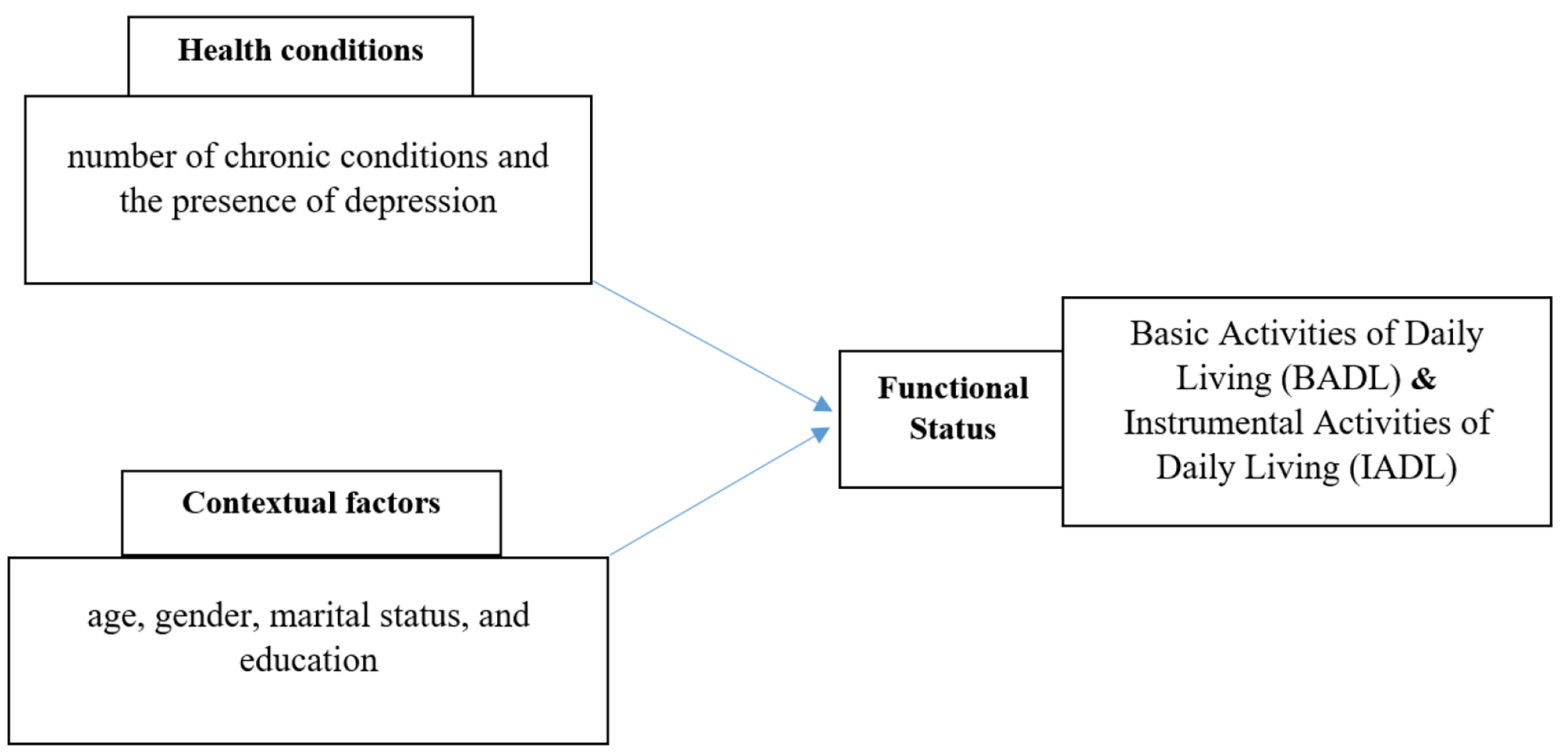
Hypothetical conceptual model

The study was guided by the World Health Organization’s ICF (International Classification of Functioning, Disability and Health) conceptual model. The ICF model conceptualizes an individual’s level of functioning as a dynamic interaction between the individual’s health conditions and contextual factors [27]. Contextual factors in the ICF guide users of the model to consider the impact of environmental factors (e.g., organizational policies) and personal factors (e.g., age, gender) on a person’s overall functioning and disability [28]. Accordingly, based on this conceptual model, we conceptualized that an individual’s health conditions (number of chronic conditions and depression), and contextual factors (age, gender, marital status, and education) predict functional status in older adults with multiple chronic conditions (Fig. 1).

The literature is also organized around the factors conceptualized as influencing functional status in the study population. The review of the literature includes studies that report findings in this regard. Female gender has been proposed as one of the predictors of functional status [29]. Explanations for gender differences in functional status are varied and reflect a complex interaction between biological and environmental factors [30]. Women suffer from more disabilities [31]. Women report experiencing more stressful life events and chronic stress in areas such as child and family health, which may translate into poorer functioning [32]. Education has also been found to predict functional status [29]. Researchers have identified that increased resource availability linked to higher education may reduce limitations related to health conditions [33]. Marital status has also been found to predict functional status. The protective role of marriage is a strong social relationship that may lead to better health and function because spouses have a key role in physical and emotional support [34]. Spouses may help with medication adherence, prepare and encourage eating healthy meals [34]. Age has also been found to adversely predict functional status [29, 35]. When people age, frailty and disability appear. Frailty is defined as a common and important aging syndrome characterized by age-related decline in physiological reserve and function of multi-organ systems, leading to increased vulnerability to adverse health outcomes and functional limitations [35, 36]. It has been found that the number of chronic conditions rather than specific conditions, or severity of conditions may is more associated with limitations in BADL and IADL [37]. Accumulation of chronic diseases in older adults increases the complexity of drug administration, and the possibility of limitations in functional status increases [38]. Depression can be among other factors that predict the functional status in older adults. Older adults with depressive symptoms may be less likely to adhere to treatment regimens, which in turn may be associated with the onset or progression of health conditions that leading to impaired functional status [39]. Also, biological changes associated with depressive symptoms, such as increased cortisol levels and insulin resistance, may increase the risks of functional status impairment [40]. Therefore, in this study, the factors included age, gender, marital status, education, number of chronic conditions, and depression were conceptualized as possible factors that can predict functional status in Iranian older adults with multiple chronic conditions.

## Methods

### Study design

This cross-sectional study was conducted from December 2022 to September 2023 at one of the largest hospitals in the north of Iran.

### Setting & sample

In the present study, older adult patients were recruited from the medical departments of one of the largest hospitals in the north of Iran. This hospital is a referral center for the province and neighboring provinces and a super-specialized center for all medical fields such as endocrinology, lung, nephrology, and rheumatology. The samples were recruited by convenience sampling. The inclusion criteria included adults aged 60 years and older, with at least two chronic conditions [41] that were assessed by self-reports, no cognitive impairment based on abbreviated mental test, consciousness and ability to communicate and desire to participate in the study. The minimum required sample size was determined as 108, based on the sample size formula for multiple regression and considering the anticipated effect size 0.15, desired statistical power level 0.8, number of predictors 8, and probability level 0.05 [42]. Totally, considering the possibility of participants with missing data, 118 older adults with multiple chronic conditions completed the questionnaires.

### Variables & data measurement

#### Demographic and health information

Patient demographic and health information was collected through self-report using a questionnaire with items on age, gender, marital status, education, number of medication, and number of chronic conditions. Age was used as a continuous variable. For the marital status, four categories including unmarried, married, divorced, and widowed were considered, and after data collection, based on frequency in each category, some categories were merged and information was presented in two categories: single (including unmarried, divorced, and widowed), and married. Education was recorded, and partitioned into three categories: high (above diploma and higher education), intermediate (diploma) and low level (illiterate or under diploma).

#### Cognition

Cognition was assessed by the Persian version of an abbreviated mental test (AMT). AMT contains 10 items which measure level of cognitive impairment. Each correct answer is scored on 0 or 1. The range of scores on this scale is between 0 and 10 [43]. The translation, validity, and reliability of the abbreviated cognition test in Iran was done by Bakhtiyari et al. (2014) [44]. The discriminant validity of AMT was appropriate for distinguishing individuals with and without cognitive impairment. Also, the sensitivity and specificity based on the diagnostic and statistical guide criteria of mental disorders were 64.9% and 64%, respectively, with a cut-off point of 7. The external reliability of the AMT was good (intergroup correlation coefficient = 0.89) and its internal reliability was acceptable (Cronbach’s alpha coefficient = 0.76) [44]. In the study sample, the internal consistency reliability of this index was acceptable (Cronbach’s alpha coefficient = 0.87).

#### Depression

Depression was measured by the Persian version of the Geriatric Depression Scale (GDS). This self-report scale contains 15 questions with yes or no response options. Each item is scored 0 or 1. The total scale score is derived, after reverse coding negatively worded items, by summing the response of the questions and can range between 0 and 15; a higher score indicates a higher level of depression [45, 46]. The translation, validity, and reliability of this questionnaire in Iran was done by Malakouti et al. (2006) [46]. In this study, the validity of the geriatric depression scale was investigated using factor analysis and criterion validity. The correlation coefficient for the 15-question form of GDS was 0.35 and 0.4 and for the 11-question form of GDS, 0.37 and 0.42 (*P* < 0.001). Also, sensitivity and specificity of 0.9 and 0.84 were calculated for GDS. Cronbach’s alpha, split-half, and test-retest coefficients for GDS were reported as 0.9, 0.85 and 0.85, respectively [46]. Internal consistency of GDS was acceptable in the study sample (Cronbach’s alpha = 0.88).

#### BADL

Basic Activities of Daily Living (BADL) was measured by the Persian version of the Katz Index of Independence in Basic Activities of Daily Living. With this Index, clients are scored yes (scored 1)/no (scored 0) for independence in each of six functions: bathing, dressing, toileting, transferring, continence, and feeding. A score of 6 indicates full function, 4 indicates moderate impairment, and 2 or less indicates severe functional impairment [47, 48]. The translation, validity, and reliability of this questionnaire in Iran was done by Azad et al. (2006) [48]. This scale demonstrated favorable construct validity with acceptable correlations with similar conceptual scales. A high to excellent correlation was found between Katz’s index of basic activities of daily living and components of movement (*P* = 0.88), self-care (*P* = 0.98), and total scores (*P* = 0.92) of the Barthel index. Also, inter-rater and intra-rater reliability of the Persian Katz index was acceptable (respectively, ICC = 0.83, ICC = 2.1, ICC = 0.93, ICC = 1.93). In an Iranian sample, the Internal consistency of this index was high (Cronbach’s alpha = 0.79) [48]. In this study, the internal consistency of this index was acceptable (Cronbach’s alpha = 0.85).

#### IADL

Instrumental Activities of Daily Living (IADL) was measured by the Persian version of the Lawton IADL scale. The scale contains 8 items that assess a person’s ability to perform 8 activities: using a telephone, shop, preparing a meal, performing housekeeping, doing laundry, using transportation, and managing medications and money. Each item is scored either 0 (lower ability) or 1 (higher ability), while the total score is 8 that indicates a complete independent functioning [49, 50]. The translation, validity, and reliability of this questionnaire in Iran was done by Hassani Mehraban et al. (2014) [51]. This scale demonstrated favorable construct validity with acceptable correlations with similar conceptual scales. The experts’ agreement on the questions was calculated using the Kruskal-Wallis table of agreement, and the χ2 values were 19.022427. The experts’ agreement indicated the content validity of all the questions (*P* < 0.05). To check inter-rater reliability, the total correlation of the test between the first and second raters (using ICC) was also very high (*P* < 0.001, *r* = 0.961), which indicates the high reliability of the test in an Iranian samples [51]. In this study sample, the internal consistency of the index was acceptable (Cronbach’s alpha = 0.81).

### Ethical considerations

After obtaining ethics permission from the university’s ethics committee (ethics code: IR.MODARES.REC.1401.108), the researcher was introduced to the research unit in a written and official manner by letter of introduction from the University of Medical Sciences. The objectives of the research were explained to the research unit and the study was conducted on those units that chose to participate in the study. Informed consent was obtained from all patients and the research unit. At first, the nurses working in the unit introduced the study to patients and got their permission to have the researcher talk to them about the study in more detail and provide more details about the study and its goals, and their rights as research participants.

### Data analysis

SPSS version 18 software [52] was used for data analysis. Descriptive statistics, including mean and standard deviation for quantitative variables and percentage for qualitative variables, were utilized. To check the normality of the quantitative variables of the study, the Kolmogorov-Smirnov test was used, which confirmed the normality of the data. Univariate linear regression analysis was used to identify a factor that may be a significant predictor for BADL and IADL. Multiple linear regression analysis was used to identify a set of predictors that may be significant predictors for BADL and IADL. By using multiple linear regression analysis, the impact of a set of independent variables on the dependent variables is investigated synchronously. In this study, the impact of each factor for predicting BADL and IADL was investigated by using univariate linear regression analysis, and also the impact of a set of factors (age, gender, marital status, education, number of chronic diseases, and depression) for predicting BADL and IADL by using multiple linear regression analysis. The tolerance and variance inflation factor (VIF) tests were used for the possibility of multicollinearity of the independent (predictor) variables. According to Menard (1955) [53], a tolerance value of less than 0.1 constitutes a certain and serious collinearity problem. Also, Myers (1990) [54] indicated that a VIF value of greater than 10 must call for a concern. The confidence interval in this study was set at 0.95. The significance level in all tests was 0.05.

## Results

### Characteristics of the participants

In this study, 118 older adults with multiple chronic conditions participated, the majority of whom were women (59.3%) and married (63.9%). The participants ranged in age from 61 to 92 years with a median age of 68 years. The median number of chronic conditions was 3. 26 (22%) of participants had at least 2 chronic conditions, and 1(0.8%) of participants had at most 6 chronic conditions, of which high blood pressure (83.1%), diabetes (63.6%), and respiratory diseases (68.6%) were the most common. The minimum and maximum number of medications in patients participating in the study were 3 and 14. Polypharmacy (using five or more medications) was found in 189% (*n* = 106) of older adult patients participating in the study. Other details are presented in Table 1.

**Table 1:** Demographic and health information of participants (*N* = 118)

Variable		Frequency (Percent) or Mean ± SD
Gender	Male	48 (40.7)
	Female	70 (59.3)
Marital status	Single	43 (36.4)
	Married	75 (63.9)
Education	Low	62 (52.5)
	Intermediate	42 (35.6)
	High	14 (11.9)
Age	Mean ± SD	70.15 ± 6.91
Number of Chronic Conditions	Two	29 (22)
	Three	36 (33.1)
	Four and more	53 (44.9)
	Mean ± SD (Total)	3.33 ± 0.94
Type of Chronic Conditions	Diabetes	75 (63.6)
	Hypertension	98 (83.1)
	Respiratory diseases	81 (68.6)
	Cardiovascular diseases	66 (55.9)
	Kidney diseases	34 (28.8)
	Rheumatism	29 (24.6)
	Other Chronic Conditions	13 (11)
Number of Medications	Mean ± SD	7.90(2.72)

The average cognitive score of participants was 9.38(0.78), which indicates that all of participants had normal cognitive status. The average depression score of participants was 10.68(2.58), which indicates that they were depressed. The average score of BADL and IADL was 2.62(1.57) and 1.40(1.06), respectively. Also, the median of BADL and IADL was 2 and 1, respectively. More than half of older adults had an BADL (50.9%) and IADL (70%) score of 2 and below, which indicates that they are dependent in performing BADL and IADL.

### The analysis of multicollinearity

The tolerance and variance inflation factor (VIF) tests were used for the possibility of multicollinearity of the independent (predictor) variables. Tables 2 and 3 showed that all of the tolerance levels are above 0.1 and also, the VIF values are below 10. Therefore, multicollinearity is not a problematic issue or concern for this study (Tables 2 and 3).

**Table 2:** Correlation coefficient matrix for the possibility of multicollinearity of the independent (predictor) variables for BADL

Model Dependent Variable: BADL	Collinearity Statistics			
	Tolerance	VIF		
(Constant)				
Marital status	0.811	1.232		
Gender	0.805	1.243		
Age	0.560	1.786		
Education	0.874	1.144		
Numbers of Chronic Conditions	0.400	2.503		
Depression	0.542	1.845		
IADL	0.698	1.433		

**Table 3:** Correlation coefficient matrix for the possibility of multicollinearity of the independent (predictor) variables for IADL

Model Dependent Variable: IADL	Collinearity Statistics	
	Tolerance	VIF
(Constant)		
Marital status	0.834	1.199
Gender	0.795	1.257
Age	0.541	1.849
Education	0.878	1.140
Numbers of Chronic Conditions	0.427	2.339
Depression	0.531	1.885
ADL	0.777	1.288

### Predictors of BADL

Based on the results of univariate linear regression analysis, age (*B*=-0.09, *P* < 0.001), gender (*B* = 0.98, *P* = 0.001), marital status (*B*=-0.69, *P* = 0.02), numbers of chronic conditions (*B*=-0.5, *P* = 0.001), depression (*B*=-0.18, *P* = 0.001) and IADL (*B* = 0.52, *P* < 0.001) were significant predictors for BADL. Based on the results of the multiple regression analysis, age (*B*=-0.04, *P* = 0.04), depression (*B*=-0.12, *P* = 0.04), and IADL (*B* = 0.46, *P* < 0.001) were significant predictors for BADL. The R-squared value of 0.39 indicates that the independent variables included in the regression model, explained 39% of the variance in BADL. Other details are provided in Table 4.

**Table 4:** Predictors of BADL using univariate and multiple regression analysis

Variable		Univariate			Multiple		
		B	Std. Error	P_value_	B	Std. Error	P_value_
Intercept					5.64	1.48	< 0.001
Gender	Male	0.98	0.28	.001^d^	0.37	0.26	0.15
	Female	~	-	-	~	-	-
Marital status	Single	− 0.69	0.29	.02^b^	− 0.05	0.27	0.83
	Married	~	-	-	~	-	-
Education	Low	− 0.74	0.46	0.11	− 0.36	0.39	0.36
	Intermediate	− 0.54	0.48	0.26	− 0.63	0.40	0.11
	High	~	-	-	~	-	-
Age		− 0.09	0.01	<.001^d^	− 0.04	0.02	.04^b^
Numbers of Chronic Conditions		− 0.5	0.14	.001^d^	− 0.34	0.19	0.08
Depression		− 0.18	0.05	.001^d^	− 0.12	0.06	.04^b^
IADL		0.52	0.07	<.001^d^	0.46	0.08	<.001^d^

a. This parameter is set to zero because it is redundant., B. Computed Using Alpha = 0.05, a: *P* ≤ 0.1, b: *P* ≤ 0.05, c: *P* ≤ 0.01, d: *P* ≤ 0.001

### Predictors of IADL

Based on the results of the univariate regression analysis, age (*B*= -0.1, *P* < 0.001), gender (*B* = 1.07, *P* < 0.001), marital status (*B*=-1.04, *P* = 0.001), numbers of chronic conditions (*B*=-0.82, *P* < 0.001), depression (B=-0.17, *P* = 0.003), and BADL (*B* = 0.58, *P* < 0.001) were significant predictors for IADL. Based on the results of the multiple regression analysis, marital status (*B*=-0.51, *P* = 0.05), numbers of chronic condition (*B*=-0.61, *P* = 0.002), and BADL (*B* = 0.46, *P* < 0.001) were significant predictor for IADL. The R-squared value of 0.45 indicates that the independent variables included in the regression model, explained 45% of the variance in IADL. Other details are provided in Table 5.

**Table 5:** Predictors of IADL using univariate and multiple regression analysis

Variable		Univariate			Multiple		
		B	Std. Error	P_value_	B	Std. Error	P_value_
Intercept					1.85	1.56	0.23
Gender	Male	1.07	0.29	<.001^d^	0.09	0.26	0.73
	Female	~	-	-	~	-	-
Marital status	Single	-1.04	0.30	.001^d^	− 0.51	0.26	.05^b^
	Married	~	-	-	~	-	-
Education	Low	− 0.28	0.48	0.56	− 0.50	0.39	0.20
	Intermediate	0.35	0.51	0.48	− 0.68	0.40	0.09
	High	~	-	-	~	-	-
Age		− 0.10	0.02	<.001^d^	− 0.01	0.02	0.59
Numbers of Chronic Conditions		− 0.82	0.14	<.001^d^	− 0.61	0.19	.002^d^
Depression		− 0.17	0.05	.003^c^	− 0.08	0.06	0.18
BADL		0.58	0.08	<.001^d^	0.46	0.08	<.001^d^

a. This parameter is set to zero because it is redundant., B. Computed Using Alpha = 0.05, a: *P* ≤ 0.1, b: *P* ≤ 0.05, c: *P* ≤ 0.01, d: *P* ≤ 0.001

## Discussion

The present study was conducted to determine the predictors of functional status in Iranian older adults with multiple chronic conditions. The results of this study showed that functional status of most Iranian older adults with multiple chronic conditions was impaired. Consistent with the results of this study, the functional status impairments in older adults ranged from about 20–46% in other studies [14, 19, 55–59], which results indicate relatively high functional status impairments in older adults. Although the results of the mentioned studies [14, 55–59] were in line with the results of this study, the impairments related to IADL in the population of this study was higher than in prior studies [14, 55–59]. Among the possible reasons for the difference between the results of this current study with those of prior studies, it is possible that the provision of healthcare, welfare, and social services in Iran are not sufficient for older adults, and Iran has faced many challenges in reaching active aging for older adults. Geriatric policies in Iran have been general and vague, while the well-being of older adults was reported to be inadequate in most of Iran’s provinces. Also, some environmental obstacles, including poor street conditions, access problems and distance to health services facilities, noise, heavy traffic, dangerous intersections, poor lighting, and cyclists on the road, may limit mobility and reduce the sense of security in older adults [60, 61]. In addition, older adults in this study had multiple chronic conditions, while other mentioned studies did not specifically focus on this population. It is possible that the cumulative burden of disease caused by the simultaneous occurrence of two or more chronic conditions may be more important than the effect of each specific disease individually, and this may lead to greater disability. On the other hand, inconsistent with the findings of this study and other aforementioned studies, in another study [62], functional disability (BADL and IADL) was reported at a low level in older adults. Among the possible reasons for the difference in the results, in a prior study [62], hospitalized and critically ill older adults were excluded from the study, while in this study and other aforementioned studies [14, 55–59], this exclusion criterion was not taken into consideration. It seems that suffering from multiple chronic conditions in older adults can threaten their independence in performing daily and instrumental activities of living and make them more functionally dependent.

In this study, it was found that age significantly predicts functional status in terms of BADL in older adults with multiple chronic conditions; this finding concurs with the those of other studies [55, 59, 63]. Therefore, it seems that with increasing age can predict functional status especially performing BADL.

In this study, depression significantly predicted functional status in terms of BADL in older adults with multiple chronic conditions which concurs with the findings of another study [64]. Another study showed that early symptoms of major depression were associated with a greater likelihood of subsequent decline in higher-level BADL compared to individuals without major depressive symptoms [65]. Depression, especially chronic and long-term depression, can damage the brain and lead to a decrease in function [66].

In this study, marital status significantly predicted IADL. Specifically, single older adults were more likely to have functional impairment in terms of performing ADL than married older adults. In line with this result in this study, it was reported in the study of Gupta et al. (2014) that functional disability was significantly significant in single or unmarried people [67]. Another study found that functional disability was significantly more seen in those participants who were widowed or separated from their spouses compared to married older adults [68].

In this study, it was found that the number of chronic diseases can predict functional status in terms of IADL in older adults with multiple chronic conditions significantly. In line with this finding in this study, it was shown in another study that older adults with chronic disease had more functional disability than older adults without chronic disease [68]. Another study reported that BADL was associated with having no chronic or disabling disease [69]. It seems that a higher number of diseases, medications and other related treatments often lead to reduced quality of life and social isolation, resulting in functional status [68].

According to the results, BADL and IADL were significant predictors for each other: IADL can predict status on performing BADL and vice versa. Consistent with the results in this study, another study [70] reported that status on IADL was the strongest predictor of BADL and vice versa. In other studies, researchers have also pointed out the significant correlation between these two concepts [71, 72]. BADL includes activities necessary for independent self-care. In contrast, IADL refers to the abilities required for an independent interaction with the environment [70]. The decline in IADL usually precedes BADL limitations and thus may serve as a predictor of future BADL decline. Conversely, autonomy in BADL serves as a precondition for autonomy in terms of IADL. Therefore, according to the results of this study and other mentioned studies, probably status on BADL/IADL can serve as a prerequisite for another related concept.

### Implications

According to the results, it seems that a comprehensive assessment of the demographic characteristics of older adults including age, gender, as well as their health conditions such as the number of chronic conditions and mental health, and more specifically their depression status can be useful for predicting the functional status of older adults. Therefore, the results showed that geriatric assessment should integrate physical and mental health care. Also, according to the results, it seems that healthcare providers can prevent or reduce functional status impairment in older adults and improve their functional status by addressing modifiable factors including number of chronic conditions and depression. In this regard, it is suggested that policymakers and health managers provide the necessary infrastructure to plan and implement interventions such as using strategies to improve mental health and lifestyle to prevent or improve depression and chronic conditions so that older adults are less at risk of functional impairment. For future studies, it is suggested to conduct the study with probability sampling. It is also suggested that researchers conduct studies among older adult populations in other different parts of Iran so that they can more accurately identify the predictors of functional status in older adults according to their specific conditions and characteristics and then adopt more effective strategies to improve it.

### Limitations

The limitation of this study is that it was conducted in the north of Iran, so its results may not be generalizable to all areas of Iran. Also, convenience sampling can limit generalizability of the findings.

## Conclusion

According to the results, older adults with multiple chronic conditions who are older, single (unmarried, divorced, widow), with a higher number of chronic conditions and depression are more at risk of impaired functional status. Therefore, this study shows the importance of the effects of demographic and health characteristics including age, gender, number of chronic conditions, and depression on the ability to perform basic and instrumental activities of daily living in older adults with multiple chronic conditions. These findings can play an important role in preventing functional status impairments or improving the functional status among older adults with chronic conditions. Nurses and other healthcare providers can use the results of this study to identify high-risk older adults.

## Data Availability

The datasets used and/or analyzed during the current study are available from the corresponding author on reasonable request.

## Abbreviations

BADL: Basic Activities of Daily Living
IADL: Instrumental Activities of Daily Living
